# CRL4A^DTL^ degrades DNA-PKcs to modulate NHEJ repair and induce genomic instability and subsequent malignant transformation

**DOI:** 10.1038/s41388-021-01690-z

**Published:** 2021-02-24

**Authors:** Maoxiao Feng, Yunshan Wang, Lei Bi, Pengju Zhang, Huaizhi Wang, Zhongxi Zhao, Jian-Hua Mao, Guangwei Wei

**Affiliations:** 1 Key Laboratory for Experimental Teratology of the Ministry of Education, Department of Cell Biology, School of Basic Medical Sciences, Cheeloo College of Medicine, Shandong University, Jinan, Shandong China; 2 Department of Clinical Laboratory, The Second Hospital, Cheeloo College of Medicine, Jinan, Shandong China; 3 Biological Systems and Engineering Division, Lawrence Berkeley National Laboratory, Berkeley, CA USA; 4 School of Preclinical Medicine, Nanjing University of Chinese Medicine, Nanjing, Jiangsu China; 5 Key Laboratory Experimental Teratology of the Ministry of Education, Department of Biochemistry and Molecular Biology, School of Basic Medical Sciences, Shandong University, Jinan, Shandong China; 6 Institute of Hepatopancreatobiliary Surgery, Chongqing General Hospital, University of Chinese Academy of Sciences, Chongqing, China; 7 School of Pharmaceutical Sciences, Shandong University, Jinan, Shandong China

**Keywords:** Non-homologous-end joining, Ubiquitylation

## Abstract

Genomic instability induced by DNA damage and improper DNA damage repair is one of the main causes of malignant transformation and tumorigenesis. DNA double strand breaks (DSBs) are the most detrimental form of DNA damage, and nonhomologous end-joining (NHEJ) mechanisms play dominant and priority roles in initiating DSB repair. A well-studied oncogene, the ubiquitin ligase Cullin 4A (CUL4A), is reported to be recruited to DSB sites in genomic DNA, but whether it regulates NHEJ mechanisms of DSB repair is unclear. Here, we discovered that the CUL4A-DTL ligase complex targeted the DNA-PKcs protein in the NHEJ repair pathway for nuclear degradation. Overexpression of either CUL4A or DTL reduced NHEJ repair efficiency and subsequently increased the accumulation of DSBs. Moreover, we demonstrated that overexpression of either CUL4A or DTL in normal cells led to genomic instability and malignant proliferation. Consistent with the in vitro findings, in human precancerous lesions, CUL4A expression gradually increased with increasing malignant tendency and was negatively correlated with DNA-PKcs and positively correlated with γ-H2AX expression. Collectively, this study provided strong evidence that the CUL4A-DTL axis increases genomic instability and enhances the subsequent malignant transformation of normal cells by inhibiting NHEJ repair. These results also suggested that CUL4A may be a prognostic marker of precancerous lesions and a potential therapeutic target in cancer.

## Introduction

Genomic stability plays a crucial role in maintaining the normal function of cells. However, genomic DNA molecules are highly susceptible to damage from a variety of factors [1]. Once DNA is damaged by external radiation, chemotherapeutic drugs, or intrinsic factors, efficient and accurate DNA damage responses will be initiated to repair damaged DNA to ensure genomic integrity [2], thereby preventing malignant transformation [3].

DNA double strand breaks (DSBs) are the most detrimental form of DNA damage [4]. NHEJ repair has a dominant and priority role in initiating DSB repair since it is not restricted by the cell cycle [5–7]. In response to DSBs, the Ku70/80 heterodimer binds to the cleaved end of DNA to recruit DNA-PKcs and then Artemis, XRCC4, Ligase IV, and XRCC4-like factor in the NHEJ repair system [8–10]. As a core member of the NHEJ repair machinery, DNA-PKcs plays an important role in maintaining genomic stability [11, 12], and cooperates with p53 to induce the apoptosis of mutant cells [13]. Thus, knocking out DNA-PKcs significantly increased tumor susceptibility in a mouse model [14, 15]. Therefore, DNA-PKcs may play an important role in the induction of malignant transformation and tumorigenesis.

The CUL4A protein belongs to the E3 ubiquitin ligase family and plays a key role in various cellular functions [16–20]. CUL4A overexpression has been detected in various types of cancers, such as breast and lung cancers, present in ≈63% of the total malignant cases, and is negatively correlated with patients’ prognosis [21]. Overexpressed CUL4A has been shown to promote the occurrence and development of tumors via several mechanisms [22–24]. Our previous studies showed that CUL4A regulates histone H3K4 methylation modification to promote tumorigenesis and metastasis [25]. In addition, other studies have shown that CUL4A inhibits the nucleotide excision repair (NER) mechanism of UV-induced DNA damage through histone ubiquitination modification and degradation of XPC, DDB2, and p21 [22, 26, 27]. Recently, CUL4A has been reported to be recruited to DNA DSB sites [28] and involved in HR repair [8], but whether it regulates the NHEJ repair process in DSB repair remains unclear. CUL4A normally exerts its oncogenic function through its substrate recognition receptors such as DTL. Previous reports indicate that DTL and CUL4A are involved in regulating p21 [29], histone H4 [30], CDT1 [31], and XPG [32] in the NER process of UV-induced DNA damage. However, whether CUL4A works with DTL to regulate the NHEJ repair pathway remains unclear.

In this study, we provided strong evidence that the CUL4A-DTL ligase complex attenuates the efficiency of NHEJ repair via the degradation of DNA-PKcs, which subsequently increases genomic instability and in turn affects the malignant transformation of normal cells.

## Results

### CUL4A inhibits DNA DSB repair through suppression of NHEJ activity by affecting DNA-PKcs

**Fig. 1:**
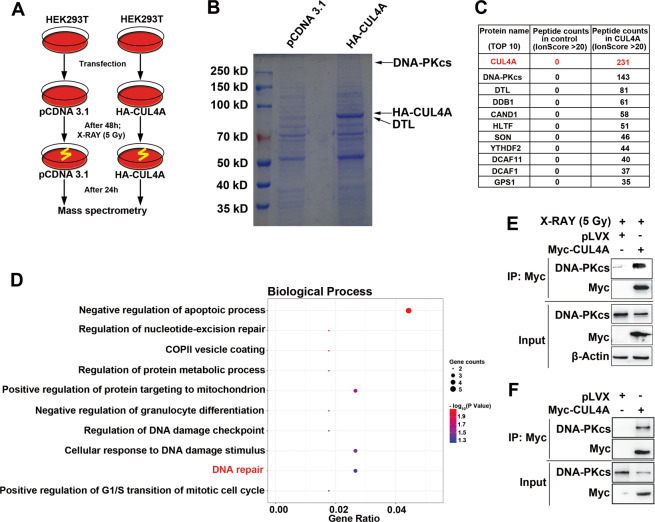
CUL4A interacts with DNA-PKcs in X-ray-irradiated cells. (**A**) Experimental flowchart for identifying CUL4A interacting proteins by mass spectrometry. (**B**) Coomassie Brilliant Blue (R250) staining of an SDA-PAGE gel containing CUL4A interacting proteins. (**C**) The top 10 abundant proteins (IonScore > 20) interacting with CUL4A identified by mass spectrometry. (**D**) Functional enrichment analysis of proteins interacting with CUL4A using the DAVID database. (**E**, **F**) HEK293T cells were transfected with Myc-CUL4A, and coimmunoprecipitation was used to detect the interaction of CUL4A and DNA-PKcs in the presence or absence of DNA damage.

To explore the role of CUL4A in DNA damage repair, HEK293T cells were transiently transfected with HA-CUL4A, and the proteins interacting with CUL4A after X-ray irradiation (IR) were enriched using an anti-HA antibody and identified by mass spectrometry (Fig. 1A, B). Mass spectrometry identified 67 proteins (Supplementary Table 1), including DCAFs (DCAF1, DTL, and DCAF11) and the substrate receptor exchange factor CAND1 [33]. The most abundant peptides identified by mass spectrometry were DNA-PKcs peptides (Fig. 1C). In addition, functional enrichment analysis revealed that the proteins in the complex with CUL4A, including DNA-PKcs (Supplementary Fig. 1A–C), a core protein in the NHEJ pathway, were involved in DNA damage repair induced by IR (Fig. 1D). The interaction between CUL4A and DNA-PKcs was further confirmed by Co-IP in HEK293T cells transfected with Myc-CUL4A in the presence or absence of DNA damage (Fig. 1E, F). To prevent the binding of DNA-PKcs to CUL4A from being mediated by genomic DNA, a nuclease (Benzonase) was added to the extracted protein lysate to digest DNA, and the results of this Co-IP also showed that CUL4A interacted with DNA-PKcs in the presence or absence of DNA damage (Supplementary Fig. 1D-F). These results suggested that CUL4A might be involved in the regulation of NHEJ repair via DNA-PKcs.

**Fig. 2:**
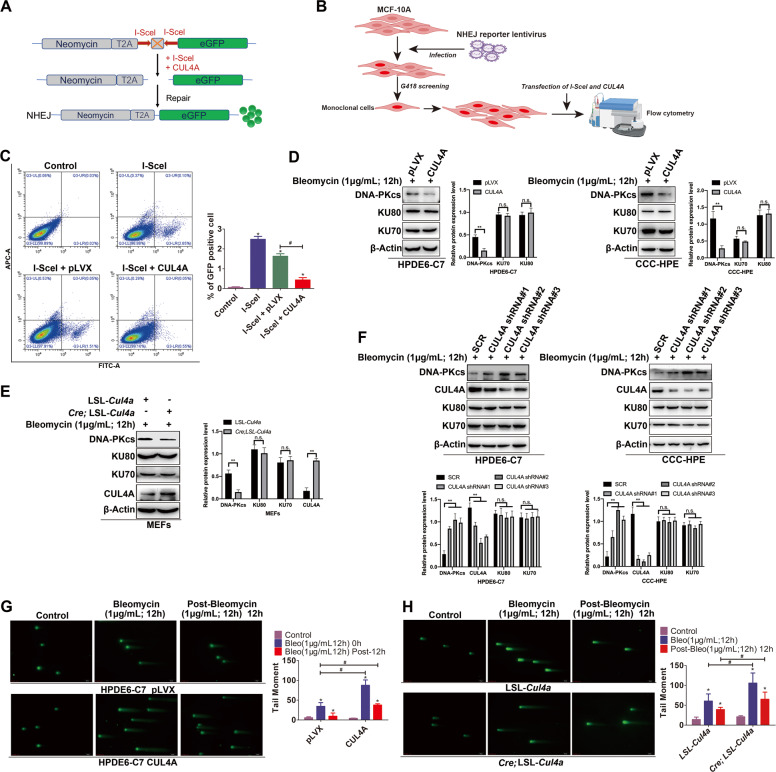
CUL4A inhibits DNA DSB repair through suppression of NHEJ activity by affecting DNA-PKcs. (**A**) Diagram of the working principle of the NHEJ reporter gene. (**B**) Schematic diagram of establishment of stable NHEJ reporter gene expression in MCF-10A cells. (**C**) With the NHEJ reporter gene system, I-SceI was used to induce DSBs, and flow cytometric analysis was used to analyze the effect of CUL4A on NHEJ efficiency. (**D**) Western blot analysis of DNA-PK kinase (DNA-PKcs, KU70/80) expression in HPDE6-C7 and CCC-HPE cells overexpressing CUL4A after DSBs induction with bleomycin. (**E**) DNA-PK protein kinase (DNA-PKcs, KU70/80) levels in MEFs from Cul4a transgenic mice after bleomycin treatment. (**F**) Expression levels of DNA-PK protein kinases (DNA-PKcs, KU70/80) in HPDE6-C7 and CCC-HPE cells with CUL4A silencing after bleomycin treatment. (**G**) Bleomycin induces DSBs, and the neutral comet assay results showed the accumulation of DSBs in HPDE6-C7 cells overexpressing CUL4A. (**H**) After treatment with bleomycin, a neutral comet assay was performed to analyze the accumulation of DSBs in MEFs from Cul4a transgenic mice. * *p* < 0.05, # *p* < 0.05, and ns = no significance based on Student’s *t*-test. The data are presented as the means ± SDs of five (**C**) or three (**D**–**H**) independent experiments. More than 100 cells were counted in **G** and **H**. The scale bars indicate 50 μm in **G** and **H**.

To verify the influence of CUL4A on NHEJ repair, we conducted an NHEJ reporter assay (Fig. 2A, B) and found that overexpression of CUL4A significantly reduced the NHEJ repair efficiency (Fig. 2C). To further investigate the mechanism by which CUL4A affected NHEJ repair, we then explored the effect of CUL4A on core proteins in the NHEJ pathway. Stable ectopic expression of CUL4A in normal pancreatic and breast epithelial cells was achieved with a lentiviral system (Supplementary Fig. 2). DSBs were induced in these cell lines with bleomycin or IR and confirmed by γ-H2AX expression (Supplementary Fig. 3). We found that the protein level of DNA-PKcs was significantly decreased by overexpression of CUL4A, while those of other NHEJ components, such as the KU70 and KU80 proteins, were not obviously affected in either the presence of DSBs (Fig. 2D; Supplementary Fig. 4A) or the absence of DSBs (Supplementary Fig. 4B). The same results were observed in *Cre*;LSL-*Cul4a* MEFs (Fig. 2E). In contrast, knocking down CUL4A expression increased the DNA-PKcs protein level after induction of DSBs (Fig. 2F). In addition, DNA-PKcs is cleaved by an active caspase after DNA damage [34]. To exclude the possibility that DNA damage activates the caspase and causes DNA-PKcs cleavage, we showed that overexpression of CUL4A did not increase the activation of caspase 3 (Supplementary Fig. 4C). These results were consistent with the inhibitory effect of CUL4A on cell apoptosis [35]. We then used a pan-caspase inhibitor (Z-YVAD-FMK) to inhibit caspase activity, and the results showed that overexpression of CUL4A also resulted in degradation of DNA-PKcs (Supplementary Fig. 4D). These results indicated that CUL4A reduced the efficiency of NHEJ repair by decreasing the DNA-PKcs protein level.

Since CUL4A suppressed NHEJ activity, we then investigated whether CUL4A can cause DSB damage accumulation. The results of neutral comet assays showed that in pancreatic epithelial cells treated with bleomycin, CUL4A overexpression significantly increased DSBs compared to that in the control group. Twelve hours after bleomycin removal, the cumulative DSB damage in CUL4A-overexpressing cells was significantly higher than that in control cells (Fig. 2G). Similar results were also found in mammary epithelial cells (Supplementary Fig. 4E). When DSBs were induced by IR, the expression of γ-H2AX in CUL4A-overexpressing cells was significantly higher than that in control cells (Supplementary Fig. 4F, G). To further confirm our findings, we treated *Cre*;LSL-*Cul4a* MEFs with bleomycin, and the results of the neutral comet assay revealed that the DNA damage in *Cre*;LSL-*Cul4a* MEFs was significantly more severe than that in control cells. Twelve hours after bleomycin removal, the DNA damage in *Cre*;LSL-*Cul4a* MEFs was still more severe than that in control cells (Fig. 2H). To further validate that the DSBs were mediated by DNA-PKcs, we overexpressed CUL4A in MCF-10A cells with silent DNA-PKcs expression. The results showed that CUL4A lost its ability to increase DSB accumulation in the silence of DNA-PKcs expression (Supplementary Fig. 4H, I).

Taken together, these results showed that CUL4A inhibited NHEJ repair activity by downregulating DNA-PKcs and subsequently led to an accumulation of DSBs in normal cells.

### CUL4A interacts with DNA-PKcs through its substrate receptor DTL

**Fig. 3:**
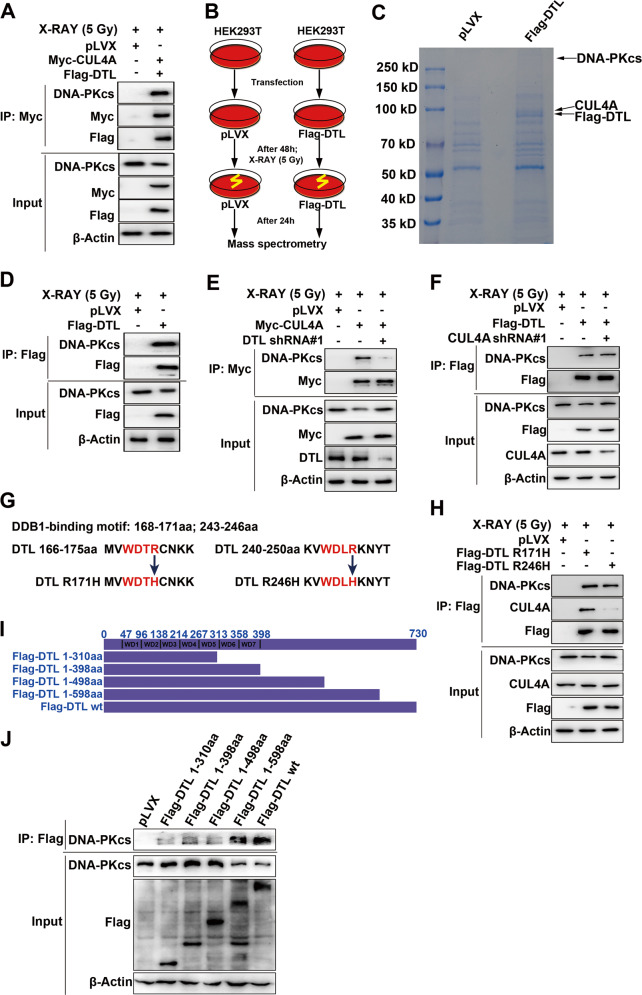
CUL4A interacts with DNA-PKcs through its substrate receptor DTL. (**A**) HEK293T cells were transfected with Myc-CUL4A and Flag-DTL, and coimmunoprecipitation was used to detect the binding of DTL to CUL4A and DNA-PKcs. (**B**) Experimental flowchart for identifying DTL interacting proteins by mass spectrometry after DSBs induction. (**C**) Coomassie Brilliant Blue (R250) staining of an SDA-PAGE gel containing DTL interacting proteins. (**D**) HEK293T cells were transfected with Flag-DTL, and coimmunoprecipitation was used to detect the binding of DTL to DNA-PKcs after IR. (**E**) Coimmunoprecipitation showed the binding of CUL4A to DNA-PKcs after silencing of DTL expression. (**F**) Coimmunoprecipitation showed the binding of DTL to DNA-PKcs after silencing of CUL4A expression. (**G**) Point mutations (R171H and R246H) in the DTL domain affected binding to DDB1. (**H**) Coimmunoprecipitation showed the binding of DTL R171H and DTL R246H to CUL4A and DNA-PKcs in HEK293T cells transfected as indicated. (**I**) Structures of DTL and its truncation mutants (**J**) Coimmunoprecipitation was used to analyze the domain mediating the binding of DTL to DNA-PKcs. All experiments were repeated independently more than three times.

We next sought to address whether CUL4A binds to DNA-PKcs through its specific substrate receptors known as DCAFs [36]. DTL is one of the most explored DCAFs and is reported to be involved in DNA repair [37]. CUL4A binds to various proteins through DTL to regulate biological processes such as DNA re-replication and UVB-induced DNA damage [38]. Furthermore, in our mass spectrometry analysis, we found that both DTL (Supplementary Fig. 1C) and DNA-PKcs were among the CUL4A binding proteins after IR. Therefore, we hypothesized that CUL4A involved in NHEJ repair through DTL. To verify our hypothesis, HEK293T cells were transiently transfected with Myc-CUL4A and Flag-DTL. Co-IP showed that DTL interacted with DNA-PKcs and CUL4A (Fig. 3A). To further confirm that CUL4A recognized DNA-PKcs via DTL, we transfected HEK293T cells with Flag-DTL, and an anti-Flag antibody was used to enrich proteins interacting with DTL after IR (Fig. 3B). CUL4A and DNA-PKcs were identified as the most abundant proteins binding to DTL by mass spectrometry analysis (Fig. 3C, Supplementary Fig. 5A, Supplementary Table 2). The Co-IP results showed that DTL bound to DNA-PKcs (Fig. 3D, Supplementary Fig. 5B-D). Moreover, silencing DTL expression impaired the binding of DNA-PKcs to CUL4A (Fig. 3E), but silencing CUL4A expression did not affect the binding of DNA-PKcs to DTL (Fig. 3F). In addition, a nuclease (Benzonase) was also added to the extracted protein lysate to digest DNA molecules, and the results of Co-IP showed that DTL also bound to DNA-PKcs (Supplementary Fig. 5E). These results indicated that CUL4A interacts with DNA-PKcs through its substrate receptor DTL.

In the UniProt database, we found that the binding site of DTL and CUL4A-DDB1 is located at aa 168–171 and aa 243–246 (https://www.uniprot.org/uniprot/Q9NZJ0). We then constructed DTL R171H and DTL R246H mutants (Fig. 3G), and Co-IP confirmed that the DTL R246H mutation significantly impaired the binding of DTL to CUL4A, while the DTL R171H mutation did not (Fig. 3H), consistent with reports indicating that R246 regulates the interaction of DTL and CUL4A-DDB1 [37, 39]. The Co-IP results also showed that neither the DTL R171H nor the R246H mutation affected the binding of DTL to DNA-PKcs (Fig. 3H). In addition, we constructed different truncation mutants of DTL (Fig. 3I), and the Co-IP results revealed that the region of DTL interacting with DNA-PKcs was located at aa 498–598 (Fig. 3J). This result further confirmed that DTL mediated the interaction between CUL4A and DNA-PKcs.

### DNA-PKcs is degraded by CRL4A^DTL^ through the ubiquitin-proteasome pathway

**Fig. 4:**
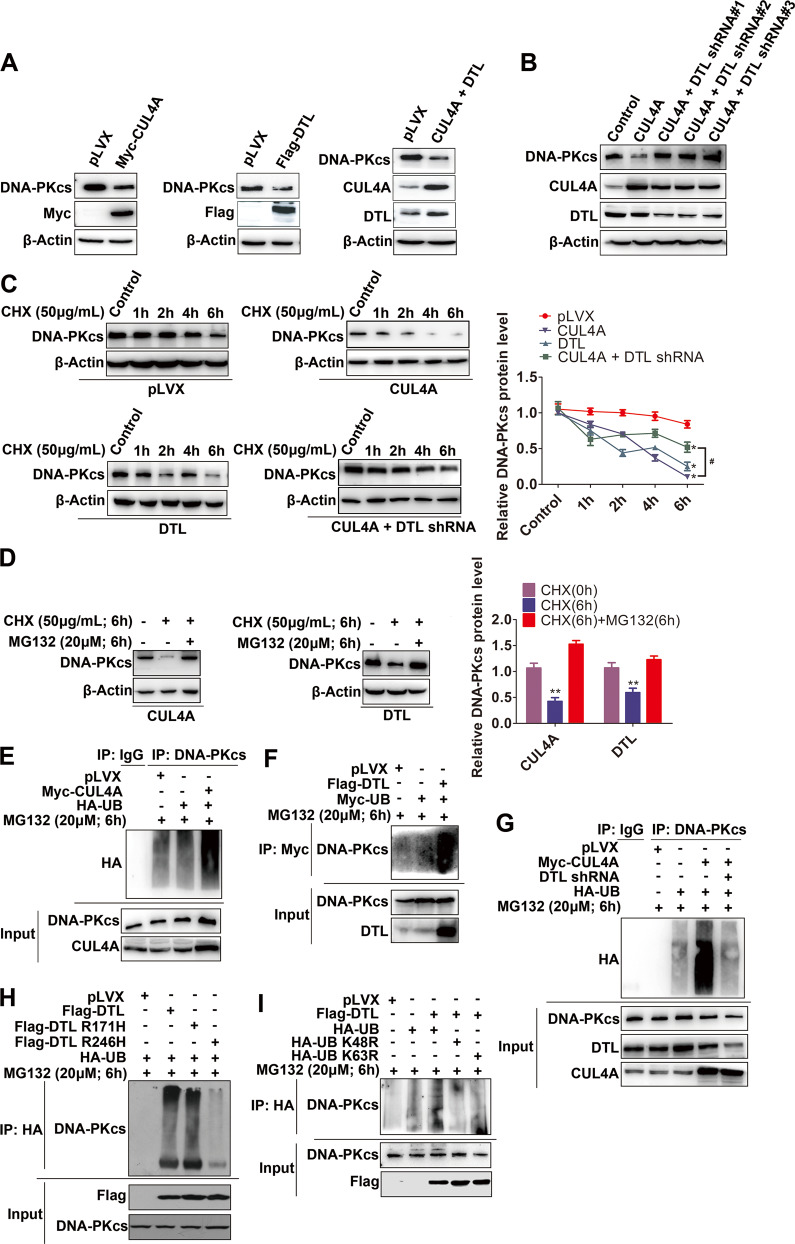
DNA-PKcs is degraded by ubiquitination through CUL4A^DTL^. (**A**) HEK293T cells were transfected with CUL4A and DTL alone or together, and the effect of CUL4A or DTL on the DNA-PKcs protein level was examined by Western blotting. (**B**) DTL was knocked out in cells overexpressing CUL4A, and the effect of CUL4A on the DNA-PKcs protein level was analyzed by Western blotting. (**C**) HEK293T cells were treated with CHX for different times, and the effect of CUL4A and DTL on the half-life of DNA-PKcs was analyzed by Western blotting. (**D**) HEK293T cells were treated with CHX and MG132 either alone or in combination, and Western blot analysis showed that CUL4A/DTL degraded DNA-PKcs via the ubiquitin-proteasome pathway. (**E**, **F**) HEK293T cells were transfected as indicated, and coimmunoprecipitation and Western blotting were used to detect the effect of CUL4A or DTL on the level of DNA-PKcs ubiquitination. (**G**) HEK293T cells were transfected as indicated, and coimmunoprecipitation and Western blotting were used to detect the effect of DTL on the ubiquitination of DNA-PKcs by CUL4A. (**H**) HEK293T cells were transfected with DTL and its point mutants (R171H and R246H), and the effects of DTL R171H and DTL R246H on DNA-PKcs ubiquitination were analyzed by coimmunoprecipitation and Western blottin. (**I**) HEK293T cells were transfected with DTL, UB, or UB mutants (K48R and K63R), and DNA-PKcs ubiquitin linkage was analyzed by coimmunoprecipitation and Western blotting. * *p* < 0.05, ** *p* < 0.01, and ^#^*p* < 0.05 based on Student’s *t*-test. The data are presented as *t*he means ± SDs of three (**C** and **D**) independent experiments.

We then evaluated whether CUL4A targets DNA-PKcs for ubiquitination and proteasomal degradation through DTL. Overexpression of CUL4A alone, DTL alone, or both CUL4A and DTL in HEK293T cells significantly decreased the DNA-PKcs protein level (Fig. 4A), while overexpression of CUL4A or DTL did not affect the DNA-PKcs mRNA level (Supplementary Fig. 6A). Significant reduction of DNA-PK kinase activity further confirmed the decrease of DNA-PKcs protein level by overexpression of CUL4A or DTL (Supplementary Fig. 6B). Silencing DTL expression abrogated the suppressive effect of CUL4A on the DNA-PKcs protein level (Fig. 4B). To clarify if CUL4A or DTL affected DNA-PKcs degradation, DNA-PKcs protein stability was analyzed in the presence of a protein synthesis inhibitor cycloheximide (CHX). As indicated in Fig. 4C, overexpression of CUL4A or DTL dramatically decreased the stability of endogenous DNA-PKcs protein. The promotion of DNA-PKcs degradation by CUL4A was reversed by silencing DTL expression (Fig. 4C). Degradation of the DNA-PKcs protein by CUL4A or DTL was restored by treatment with proteasome inhibitors (Fig. 4D). Collectively, these results indicated that CUL4A promoted proteasomal degradation of DNA-PKcs through DTL.

We then examined whether CUL4A promotes the ubiquitination of DNA-PKcs through DTL. The IP results showed that overexpression of CUL4A or DTL increased the ubiquitination of endogenous DNA-PKcs (Fig. 4E, F). Silencing DTL expression reversed the increase in DNA-PKcs ubiquitination induced by CUL4A (Fig. 4G). As expected, we found that overexpression of mutant DTL R246H did not affect the level of DNA-PKcs ubiquitination (Fig. 4H). Additionally, we found that CRL4A^DTL^ ubiquitinated DNA-PKcs via K48-linked ubiquitination (Fig. 4I). Collectively, these results indicated that CUL4A promoted DNA-PKcs ubiquitination and proteasomal degradation through its substrate receptor DTL.

### DTL suppresses NHEJ activity to increase DNA DSB accumulation

**Fig. 5:**
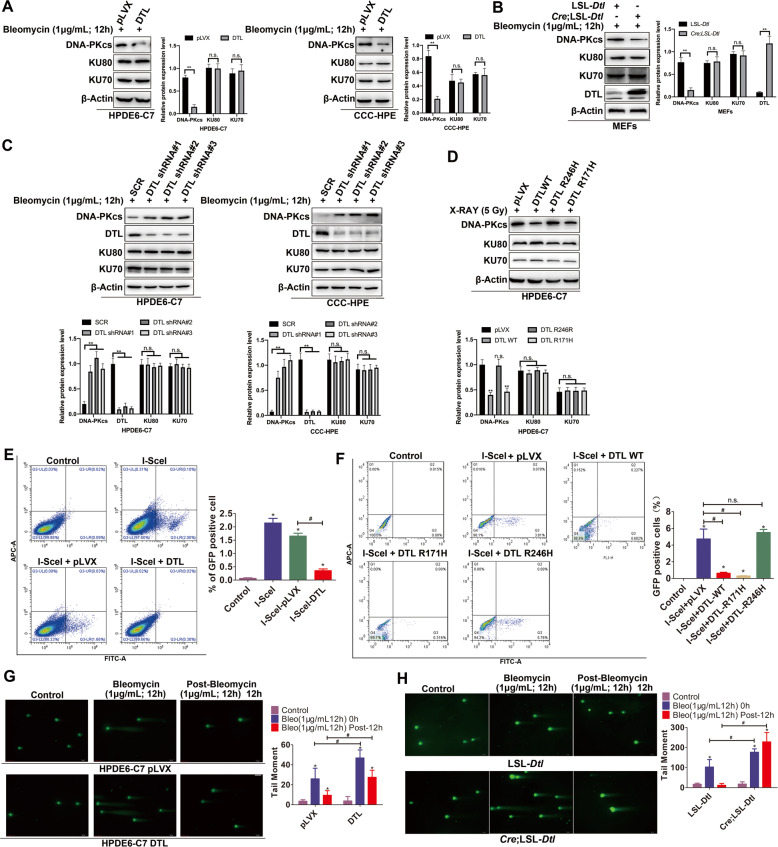
DTL reduces NHEJ activity to increase DNA DSBs. (**A**) Western blot analysis of DNA-PK kinase (DNA-PKcs, KU70/80) expression in HPDE6-C7 and CCC-HPE cells overexpressing DTL after DSBs induction with bleomycin. (**B**) The effect of DTL on the expression of DNA-PK protein kinases (DNA-PKcs, KU70/80) in MEFs from *Dtl* transgenic mice. (**C**) The expression of DNA-PK protein kinases (DNA-PKcs, KU70/80) in HPDE6-C7 and CCC-HPE cells with DTL silencing. (**D**) The DNA-PKcs protein level was detected by Western blotting in HPDE6-C7 cells stably overexpressing DTL and its point mutants (R246H and R171H) after IR. (**E**, **F**) With the NHEJ reporter gene system, I-SceI was used to induce DSBs, and flow cytometry was used to analyze the effect of DTL and its point mutants (R246H and R171H) on NHEJ efficiency. (**G**) After treatment with bleomycin, a neutral comet assay was performed to analyze the accumulation of DSBs in HPDE6-C7 cells overexpressing DTL (**H**) After treatment with bleomycin, a neutral comet assay was performed to analyze the accumulation of DSBs in MEFs from *Dtl* transgenic mice. * *p* < 0.05, # *p* < 0.05, and ns = not significant based on Student’s *t*-test. The data are presented as the means ± SDs of five (**E** and **F**) or three (**A**–**D**) independent experiments. The scale bars indicate 50 μm in **G** and **H**.

Since we showed that the CUL4A-DTL ligase complex regulates DNA-PKcs, we hypothesized that DTL has a function similar to that of CUL4A in the NHEJ pathway. Normal pancreatic and breast epithelial cell lines with ectopic DTL expression were generated (Supplementary Fig. 2). Among the core components of the NHEJ pathway, DTL specifically decreased the DNA-PKcs protein level under the pressure of DSB damage (Fig. 5A; Supplementary Fig. 7A). The same results were observed in *Cre*;LSL-*Dtl* MEFs (Fig. 5B). Silencing DTL expression restored the DNA-PKcs protein level (Fig. 5C). The inhibitory effect of the DTL R246H mutation, which disrupted the binding of DTL to the CUL4A-DDB1 complex, on the DNA-PKcs protein level was abolished (Fig. 5D). Experiments in the NHEJ reporter system (Supplementary Fig. 7B) confirmed that overexpression of DTL significantly reduced NHEJ repair efficiency (Fig. 5E), while the DTL R246H mutation did not (Fig. 5F). Neutral comet experiments showed that in pancreatic epithelial cells treated with bleomycin, DSBs were significantly increased in the DTL overexpression group compared to the control group. Twelve hours after bleomycin removal, significantly more DSBs had accumulated in the DTL overexpression group than in the control group (Fig. 5G). Similar results were also found in mammary epithelial cells (Supplementary Fig. 7C). The expression of γ-H2AX in DTL-overexpressing cells was significantly higher than that in control cells after the induction of DSBs (Supplementary Fig. 7D, E). When DSBs were induced with bleomycin, neutral comet experiments revealed that the DNA damage in *Cre*;LSL-*Dtl* MEFs was significantly more severe than that in control cells. Twelve hours after bleomycin removal, the DNA damage in *Cre*;LSL-*Dtl* MEFs was still higher than that in control cells (Fig. 5H). Finally, a stable expression of DTL was established in MCF-10A cells with DNA-PKcs knockout, and the results showed that overexpression of DTL did not increase DSB accumulation induced by bleomycin (Supplementary Fig. 7F, G). These results clearly demonstrated that DTL, like CUL4A, inhibited NHEJ activity by degrading DNA-PKcs and led to an increase in DSBs in normal cells, further confirming the role of DTL in mediating the regulation of NHEJ activity by CUL4A.

### CRL4A^DTL^ is recruited to DSB sites and degrades DNA-PKcs in the nucleus

**Fig. 6:**
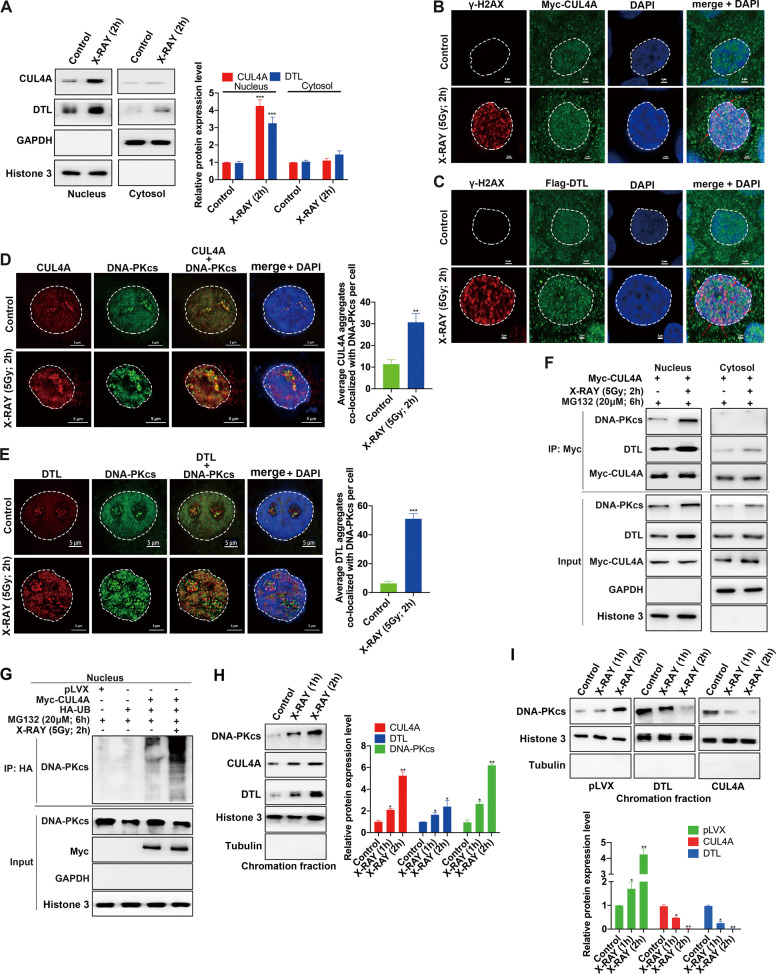
CRL4A^DTL^ is recruited to DSB sites and degrades DNA-PKcs in the nucleus. (**A**) Nuclear and cytoplasmic protein fractionation assays showed the intracellular localization of CUL4A and DTL after DSB induction in HPDE6-C7 cells. (**B**, **C**) Immunofluorescence experiments showed the colocalization of CUL4A and DTL with γ-H2AX after DSB induction in HPDE6-C7 cells. (**D**, **E**) Immunofluorescence experiments showed the colocalization of CUL4A and DTL with DNA-PKcs after DSB induction in HPDE6-C7 cells. (**F**) Nuclear and cytoplasmic protein fractionation assays and coimmunoprecipitation showed the intracellular binding sites of CUL4A and DNA-PKcs. (**G**) Nuclear protein fractionation assays and coimmunoprecipitation showed that CUL4A ubiquitinated DNA-PKcs in the nucleus after DSB induction (**H**, **I**) A chromatin fractionation assay was performed to show the changes in CUL4A, DTL, and DNA-PKcs in chromatin after DSB induction in HPDE6-C7 cells. * *p* < 0.05, ** *p* < 0.01, *** *p* < 0.001 based on Student’s *t*-test. The data are presented as *t*he means ± SDs of five (**A**) or three (**D**, **E**, **H**, and **I**) independent experiments. The scale bars indicate 2 μm in **B** and **C**, and 5 μm in **D** and **E**.

To further understand more details about how CUL4A and DTL are involved in the regulation of NHEJ activity, we analyzed the changes in CUL4A and DTL localization after the induction of DSBs. The results of nuclear and cytoplasmic protein fractionation assays indicated that CUL4A and DTL clearly aggregated in the nucleus after DSB induction (Fig. 6A) and colocalized with γ-H2AX (Fig. 6B, C). Simultaneously, the results of immunofluorescence and IP experiments revealed that the location where CUL4A or DTL interacted with the DNA-PKcs protein was mainly in the nucleoli (Fig. 6D, E) and that their binding ability increased after DSB induction (Fig. 6F). Furthermore, CUL4A ubiquitinated DNA-PKcs in the nucleus, and the ubiquitination of DNA-PKcs was increased after DSB induction (Fig. 6G). These results indicated after DSB induction, CUL4A and DTL aggregated in the nucleus where they ubiquitinated DNA-PKcs and led to DNA-PKcs degradation. Interestingly, the chromatin fractionation assay results revealed that when DSBs were present, CUL4A, DTL and DNA-PKcs aggregated on chromatin (Fig. 6H). However, we found that, in the absence of proteasome inhibitor, overexpression of CUL4A or DTL significantly reduced the binding of DNA-PKcs to chromatin (Fig. 6I). This pattern indicated that the reduced level of DNA-PKcs binding to chromatin was most likely due to the ubiquitination and degradation of DNA-PKcs by CUL4A or DTL. Consequently, the decrease in the binding of DNA-PKcs to chromatin led to a decrease in the efficiency of NHEJ repair.

### CUL4A increases genomic instability and enhances subsequent malignant transformation

**Fig. 7:**
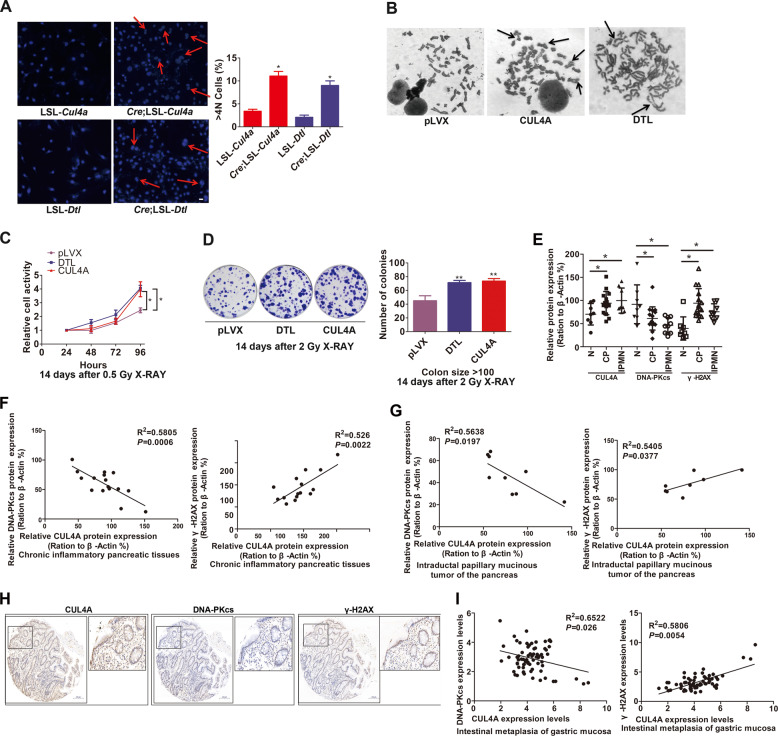
CUL4A increases genomic instability and enhances subsequent malignant transformation. (**A**) Hoechst staining of *Cre*;LSL-*Cul4a* and *Cre*;LSL-*Dtl* MEF nuclei to show multinucleated cells. (**B**) Chromosome morphology analysis showed the effect of CUL4A and DTL on the chromosome structure in HPDE6-C7 cells. (**C**, **D**) Fourteen days after IR, MTT, and colony formation assays were used to examine the effect of CUL4A and DTL on the proliferative potential of HPDE6-C7 cells. (**E**) Histogram showing the statistical analysis of the protein expression levels of CUL4A, DNA-PKcs, and γ-H2AX in pancreatic tissue (N), chronic pancreatitis tissue (CP), and intraductal papillary mucinous neoplasm (IPMN) tissue, as detected by Western blotting. (**F**, **G**) Correlation analysis between CUL4A and DNA-PKcs or γ-H2AX in chronic pancreatitis tissues or intraductal papillary mucinous neoplasm tissues, respectively. (**H**) Immunohistochemical analysis of CUL4A, DNA-PKcs, and γ-H2AX protein expression levels in intestinal metaplasias of the gastric mucosa. (**I**) Correlation analysis between CUL4A and DNA-PKcs or γ-H2AX in intestinal metaplasias of the gastric mucosa. * *p* < 0.05 and ** *p* < 0.01 based on Student’s *t*-test in **C** and **E** and correlation test in **F**, **G**, and **I**. The data are presented as the means ± SDs of three (**A**) independent experiments. The scale bars indicate 25 μm in **A**.

As DNA DSB damage eventually results in genomic instability and induce malignant transformation of normal cells [40–42], we then investigated whether CUL4A can affect genomic stability and malignant transformation of normal cells through affecting DNA damage repair. Hoechst staining of *Cre*;LSL-*Cul4a* and *Cre*;LSL-*Dtl* MEF nuclei showed that CUL4A or DTL increased the number of multinucleated cells (Fig. 7A). Karyotype analysis further revealed deletion and distortion of morphological changes in chromatin in HPDE6-C7 cells overexpressing CUL4A or DTL (Fig. 7B). Genomic instability is closely related to malignant transformation of normal tissues or cells. To explore the effect of CUL4A^DTL^ on malignant transformation of normal cells, HPDE6-C7 cells were exposed to IR and cultured for 14 days to obtain monoclonal cells, and the Western blot analysis results indicated that CUL4A^DTL^ still negatively regulated the DNA-PKcs protein level (Supplementary Fig. 8). Functional analysis showed that overexpression of CUL4A or DTL significantly increased the proliferative potential of HPDE6-C7 cells after IR (Fig. 7C, D).

To further clarify the relationship between CUL4A and malignant transformation, we examined the expression patterns of CUL4A from normal to precancerous lesions in human pancreatic tissues. With the increase in the malignant tendency of pancreatic tissues from normal to chronically inflamed to intraductal papillary mucinous tumors, the CUL4A level gradually increased. Moreover, the DNA-PKcs level decreased, and the γ-H2AX level increased (Fig. 7E, Supplementary Fig. 9). The negative correlation between CUL4A and DNA-PKcs levels and the positive correlation between CUL4A and γ-H2AX levels were consistent with our in vitro findings (Fig. 7F, G). A negative correlation between CUL4A and DNA-PKcs levels and a positive correlation between CUL4A and γ-H2AX levels in precancerous gastric lesions (intestinal metaplasia of gastric mucosa) were also found (Fig. 7H, I). These clinical findings further confirmed our conclusions that CUL4A decreased the DNA-PKcs level to inhibit DSB repair and increased genomic instability and malignant transformation.

## Discussion

**Fig. 8:**
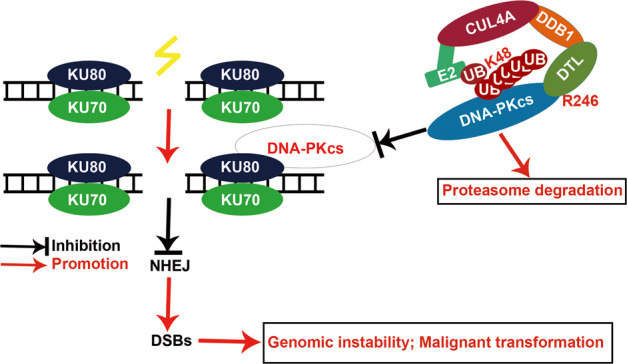
Working model. CUL4A, through DTL-specific recognition and ubiquitination of DNA-PKcs, affects the NHEJ repair pathway to increase cell genomic instability and enhance subsequent malignant transformation of normal cells.

CUL4A, an E3 ubiquitin ligase, recognizes, ubiquitinates, and degrades proteins to regulate multiple physiological processes, including DNA replication and some DNA repair processes, such as NER and HR [38]. However, all these processes mainly occur in the S or G2 phases of the cell cycle, and there are few reports on the function of CUL4A in NHEJ, a major DNA damage repair process executed throughout the cell cycle [6]. In this study, we reported a novel function of CUL4A in regulating the NHEJ mechanism and thus promoting genomic instability and malignant transformation of normal cells by degrading DNA-PKcs through its substrate receptor DTL (Fig. 8).

The functions of CUL4A in regulating genomic stability reported to date are cell cycle dependent, and different DCAFs (DDB1 and CUL4A-associated factors) and substrates are believed to define the specific function of CUL4A. During normal DNA replication, DDB1-CUL4A-DTL degrades Cdt1, p21, and Set8 by ubiquitin modification to prevent re-replication to maintain genomic stability in the S phase of the cell cycle [43–46]. CUL4A-DDB1 targets Chk1 to enable faithful resumption of DNA replication and cell cycle progression following recovery from replicative stress and DNA damage [47]. During DNA damage, CUL4A-DDB1 is recruited to DSB sites and promotes HR through ubiquitination of RECQL4, causing it to aggregate at DSBs to perform its phosphorylation function in the S and G2 phases [8, 28]. DDB1-CUL4A-Wdr70-mediated monoubiquitination of H2B promotes DNA terminal splicing during HR [48]. However, in *Cul4a*^-/-^ MEFs, knockout of Cul4a was found to block cells from entering S phase, increase NER repair, maintain genomic stability, and resist skin carcinogenesis caused by UV [22]. The above findings suggest that CUL4A recognizes certain substrates through different DCAFs to participate in regulating genomic stability and that CUL4A-mediated maintenance of genomic stability is closely related to its expression level in different phases of the cell cycle after DNA damage. To better understand the differential mechanism of CUL4A in DNA repair, we used mass spectrometry to identify proteins that bind to CUL4A after cells were subjected to IR. Functional enrichment analysis of these proteins binding to CUL4A indicated that CUL4A was indeed involved in DNA repair after IR and that in addition to DTL, DNA-PKcs, a core component of the cell cycle-independent NHEJ pathway, was the most frequently detected protein among the proteins that interact with CUL4A. Subsequent comprehensive analysis confirmed that CRL4A^DTL^, by degrading DNA-PKcs and thereby inhibiting the NHEJ mechanism, increased the accumulation of DSBs and promoted genomic instability in models of DSBs induced by bleomycin and IR, which are reported to induce DSBs in an S phase-independent manner [49].

When DSBs occur, a precision and fine-tuned machinery is activated for competitive selection of NHEJ and HR to repair these DSBs. DNA-PKcs is reported to be involved in the selection of NHEJ or HR for DSB repair [50]. In general, activation/phosphorylation of DNA-PKcs enables inactivation/mutation of NHEJ and DNA-PKcs, in turn facilitating the HR mechanism. In the S and G2 phases of the cell cycle, BRCA1 interacts with DNA-PKcs to inhibit its autophosphorylation and favor HR [51]. However, the mechanism underlying the selection of HR or NHEJ by DNA-PKcs to repair DSBs is still controversial and might be more complicated than currently believed. It has been reported that knockout or pharmacological inhibition of DNA-PKcs causes HR impairment [52, 53]. In addition, research has found that after DSB induction, DNA-PKcs promotes hyperphosphorylation of RPA, causing the RPA-p53 complex to dissociate, allowing RPA to bind to ssDNA, and promoting HR through RAD51 after DSB induction [50, 54]. Unexpectedly, more experiments showed that after knockout of DNA-PKcs, RPA hyperphosphorylation was inhibited, abolishing RPA-p53 dissociation and inhibiting the binding of RPA to ssDNA, which further impaired HR [53]. It is possible that degradation of DNA-PKcs by CRL4A^DTL^ in our study may have inhibited RPA hyperphosphorylation and then affected the binding of RPA to ssDNA and impaired HR. This possibility may explain why the DSB damage was not repaired by HR and accumulated when DNA-PKcs was degraded by CUL4A. In summary, there are two explanations: (1) CUL4A or DTL inhibits the NHEJ repair pathway by degrading DNA-PKcs and then increases HR repair pathway activity in normal cells; (2) CUL4A degrades DNA-PKcs by ubiquitination and inhibits the activity of HR-related proteins (such as RAP-p53). Further experiments are essential to reveal how DSB damage cannot be repaired under the conditions of CUL4A and/or DTL overexpression.

In addition, evidence continues to accrue that alternative NHEJ pathway also contribute to DNA DSB repair, especially in cancerous cells through a distinct mechanism [55, 56]. When canonical NHEJ is suppressed, especially when Ku proteins are inhibited as Ku proteins are showed to suppress alternative NHEJ, alternative NHEJ will be activated to repair DSBs [57, 58]. However, CUL4A/DTL mainly degrade DNA-PKcs and Ku proteins are not affected, whether alternative NHEJ is activated in this situation needs further experimental verification.

Deregulated DNA-PKcs activity is closely related to many tumors. In our study, the expression of DNA-PKcs in human precancerous tissues was significantly lower than that in normal tissues and decreased with the increase in the malignancy of precancerous pancreatic tissues. In addition, there was a negative correlation between DNA-PKcs and γ-H2AX levels in the precancerous lesions in pancreatic, intestinal and gastric cancer tissues. The results of these clinical tissue analysis are consistent with the reported function of DNA-PKcs in tumorigenesis. Research on *Dna-pkcs*^-/-^ mice found that deletion of DNA-PKcs leads to tumor susceptibility [14, 59]. Other studies also found that intestinal hyperplastic polypoid lesions, differentiated colonic epithelial cells, aberrant crypt foci and thymic lymphomas were induced in *Dna-pkcs*^-/-^ mice [60]. These results suggested that depletion of DNA-PKcs may have a potential role in tumorigenesis. However, the role of DNA-PKcs in tumorigenesis is not fully understood. Our report that degradation of DNA-PKcs by CRL4A^DTL^ impairs DSB repair, resulting in genomic instability, may shed light on the role of DNA-PKcs in tumorigenesis. However, the importance of DNA-PKcs in cancer progression and metastasis is still controversial. Clinical evaluation has shown that DNA-PKcs is significantly increased in some types of tumors, such as prostate, lung, and hepatocellular tumors, and that DNA-PKcs dysregulation is closely related to the development of distant metastasis and reduced survival in prostatic cancer and melanoma [61]. In addition, overexpression of DNA-PKcs is closely related to radiation resistance in a variety of tumors, such as thyroid [62] and uterine [63] cancers. Contrasting findings have been reported in gastric, breast, cervical, lung, and pancreatic cancer analyses. In our preliminary analysis, we did not observe any significant indications in breast and pancreatic cancer cell lines and tissues as observed above in normal breast and pancreatic cell lines and MEFs. More complex and intricate programs regulating DNA-PK function during tumor progression may exist, and intensive and meticulous investigation of DNA-PKcs function is necessary.

In summary, we revealed DNA-PKcs, a key component in the NHEJ mechanism, as a newly identified target protein of the CUL4A E3 ubiquitin ligase. Moreover, CUL4A reduces NHEJ repair efficiency by degrading DNA-PKcs throughout the cell cycle, increasing genomic instability and subsequent malignant transformation. Our research may provide a new mechanism of CUL4A in tumorigenesis, which may provide both a new target for the diagnosis of precancerous lesions in the clinic and a new treatment strategy for precancerous lesions. Finally, we will continue to explore other related mechanisms of CUL4A and its DCAFs in tumorigenesis and enhance the understanding of the mechanisms by which CUL4A mediates tumorigenesis.

## Materials and methods

### Expression plasmids

The wild-type HA-CUL4A and Flag-DTL plasmids were previously constructed by our lab. The Myc-CUL4A and Flag-DTL lentiviral vectors were subsequently constructed. The shRNA sequences targeting CUL4A were as follows: CUL4A shRNA #1, CACCTATGTGCTGCAGAACTC, CUL4A shRNA #2, TGATCATGATCAGAAGCATCT, and CUL4A shRNA #3, CAGGCACAGATCCTTCCGTTT. The shRNA sequences targeting DTL were as follows: DTL shRNA #1, gcCTAGTAACAGTAACGAGTA, DTL shRNA #2, ctGGTGAACTTAAACTTGTTA, and DTL shRNA #3, gcTCCCAATATGGAACATGTA. The pDRR plasmid was purchased from Addgene.

### Mass spectrometry

HEK293T cells were transfected with HA-CUL4A or Flag-DTL and irradiated with X-rays (5 Gy) 48 h later. After 24 h, the anti-HA or anti-Flag antibody was used to enrich proteins binding to CUL4A or DTL. SDS-PAGE was performed with Coomassie blue, and the binding proteins were identified by mass spectrometry (Maxis II, Advanced Medical Research Institute, Shandong University). Mascot software was used to identify protein names, and the binding proteins were required to contain more than two high-fidelity peptides (IonScore > 20).

### Neutral comet assay

Cells were treated with bleomycin for 12 h and were then collected and resuspended in PBS (Ca^2+^- and Mg^2+^-free) at a concentration of 1 × 10^5^ cells/ml. Comet assays were then performed according to the reagent kit (Trevigen, MD, USA) instructions. Cellular DNA damage was analyzed with the software CometScore.

### Irradiation

Cells were irradiated using a 2D image-guided precision X-ray biological irradiator (X-RAD 225 OptiMAX, Advanced Medical Research Institute, Shandong University). The radiation dose rate was 2 Gy/min.

### NHEJ activity assay

The MCF-10A cell line stably expressing the DSB NEHJ Repair reporter (Addgene, Catalog #98895) was constructed as reported in the literature [64]. DSBs were induced by the transfection of I-Sce1 and repaired by NHEJ, leading to GFP expression. The results were obtained by flow cytometric analysis.

### Statistical analysis

All statistical analyses were performed with Statistical Product and Service Solutions (SPSS) software. Student’s *t-*test and one-way analysis of variance were used to evaluate differences in statistical data. Data are presented as the mean ± SD values, and *p* < 0.05 was considered to indicate a significant difference.

More information of the materials and methods is in the Supplementary Materials.

## Supplementary information

Supplementary materials
