# Supplementary material for: CRL4ADTL degrades DNA-PKcs to modulate NHEJ repair and induce genomic instability and subsequent malignant transformation
Source: Oncogene. 2021 Feb 24;40(11):2096–111. doi: 10.1038/s41388-021-01690-z (PMC7979543; doi:10.1038/s41388-021-01690-z)
Supplement: Supplementary file 1 — Supplementary materials [file 41388_2021_1690_MOESM1_ESM.doc]

**Supplementary materials and methods**

**Reagents and antibodies**

Bleomycin was purchased from Enzo Biochem (New York City, New York, USA). Cycloheximide (CHX) and MG132 were purchased from Calbiochem (Darmstadt, Germany). Z-YVAD-FMK was purchased from MedChemExpress (New Jersey, USA). Benzonase was purchased from Merck (New Jersey, USA). A protease inhibitor cocktail was purchased from Roche (Basel, Switzerland). RIPA buffer and nuclear and cytoplasmic protein extraction kits were purchased from Beyotime Biotechnology (Beijing, China). Antibodies against DNA-PKcs (ab32566), KU70 (ab92450), KU80 (ab80592), γ-H2AX (ab26350) and CUL4A (ab72548) were purchased from Abcam (Cambridge, United Kingdom). Antibodies against HA (h3663) and Flag (F1804) were purchased from Sigma. Antibodies against histone 3 (#4499), Myc (#2276), caspase-3 (#9665), GAPDH (#5174), β-Actin (#3700), Alexa Fluor 488 (#8878) and Alexa Fluor 555 (#8953) were purchased from Cell Signaling Technology (Boston, Massachusetts, USA). The anti-DTL antibody was purchased from Novus Biologicals (Colorado, USA).

**Culture of cell lines and mouse embryonic fibroblasts (MEFs)**

MCF-10A normal human breast cells were cultured in Dulbecco’s modified Eagle’s medium (DMEM)/F12 supplemented with 5% horse serum (Biological Industries, Israel), 10 μg/ml insulin (Solarbio), 20 ng/ml epidermal growth factor (Pepro Tech, Cranbury, New Jersey, USA), 100 ng/ml cholera toxin (Macgene, Beijing, China), 0.5 μg/ml hydrocortisone (Macgene) and 10% penicillin and streptomycin (Thermo Fisher Scientific). Normal human breast cells (HBL-100) and normal human pancreatic cells (HPDE6-C7 and CCC-HPE) were grown in DMEM supplemented with 10% fetal bovine serum (Biological Industries) and 10% penicillin and streptomycin (Thermo Fisher Scientific, Waltham, Massachusetts, USA). LSL-*Cul4a* or LSL-*Dtl* MEFs were isolated from embryonic day (E) 14 embryos and cultured in DMEM supplemented with 10% fetal bovine serum and 10% penicillin and streptomycin. To establish stable expression of *Cul4a* or *Dtl* in MEFs, LSL-*Cul4a* or LSL-*Dtl* MEFs were infected with adenovirus expressing the Cre gene. All cells were grown at 37°C in 5% CO_2_ and under saturated humidity conditions. LSL-*Cul4a* mice were generated as described [1], and the same vector and procedure were used to generate LSL-*Dtl* mice.

**Western blot and immunoprecipitation**

Cells were lysed in RIPA lysis buffer supplemented with protease inhibitor cocktail and Benzonase for 30 min at 4°C. Cellular debris was removed by centrifugation at 12000 rpm for 25 min. During immunoprecipitation (IP), the cell lysate was first incubated with antibodies against HA, DNA-PKcs, and Flag overnight at 4°C and then with Protein A/G agarose beads (Merck-Millipore, Darmstadt, Germany) for 4 h at 4°C. The protein A/G agarose beads were washed three times with PBS containing 1% Tween 20 (Solarbio, Beijing, China) and boiled with SDS loading buffer at 97°C for 5 min. The samples were then subjected to SDS-PAGE, transfected and incubated with different antibodies.

**Cell proliferation**

Colony formation, MTT and soft agar assays to assess cell proliferation were performed as previously described [2, 3].

**Immunostaining**

Cells were cultured on coverslips and treated with X-rays. Twelve hours after treatment, cells were fixed with 4% paraformaldehyde for 8 min and washed three times with PBS. Cells were then permeabilized with 0.4% Triton X100 in PBS for 15 min, washed three times with PBS, incubated with 5% blocking solution for 40 min, and washed three more times with PBS. Coverslips were incubated with different antibodies overnight at 4°C and were then washed three times with PBS, incubated with different immunofluorescent secondary antibodies for 1 hour at room temperature and washed three more times with PBS. Coverslips were then stained with DAPI for 5 minutes and washed three times with PBS. Coverslips were mounted on a glass slide with an anti-fluorescence quencher, and images were acquired with a Zeiss LSM780 confocal microscope.

**Karyotype analysis**

Cells were treated with 0.8 μg/ml colchicine for 4 h at 37°C. Cells were collected by centrifugation to remove the supernatant and were then treated with 25 mM KCl for 20 min at 37°C. The hypotonic solution was removed by centrifugation, and cells were fixed with an isopropyl alcohol-methanol fixative for 15 min. The isopropanol-methanol fixative was removed by centrifugation, and the cell suspension was added dropwise to a glass slide. Cells were stained with Giemsa for 15 min, washed with water and dried. The cellular chromosomes were observed with a microscope (Olympus DP72).

**Detection of cell aneuploidy**

Cells were fixed in 70% ethanol and washed with PBS. Cells were then incubated with 1 μg/ml Hoechst (Solarbio) in PBS for 30 min in the dark. Cells were observed using a fluorescence microscope (Olympus DP72).

**Hight salt protein extraction and DNA-PK kinase assay**

High-salt extraction of cell lysate was performed as described by BB Olsen et al [4]. Briefly, the collected cells were washed and incubated in a low-salt buffer [10 mM HEPES (pH 7.4), 25 mM KCl, 10 mM NaCl, 1.1 mM MgCl2, 0.1 mM EDTA, and 0.1 mM DTT] containing protease inhibitor cocktail (Roche, Basel, Switzerland) and 100 nM okadaic acid on ice for 5 minutes followed by another 5 minutes’s incubation in a dry ice/ethanol bath. Cells were quickly thawed in a 37°C water bath and high-salt extraction buffer (5 M NaCl, 100 mM MgCl2, and 10 mM DTT) was then added. After incubation on ice for 5 minutes, the pellet was washed with high-salt wash buffer [10 mM HEPES (pH 7.4), 25 mM KCl, 0.5 M NaCl, 10 mM MgCl2, 0.1 mM EDTA, and 1 mM DTT]. DNA-PK kinase activity was detected with ADP-Glo^TM^ Kinase Assay (V9101, Promega, Madison, Wisconsin, USA) and DNA-PK Kinase Enzyme System (V4106, Promega, Madison, Wisconsin, USA) kits, described briefly as follows: Add the protease inhibitor cocktail, high-salt cell extract and substrate/ATP mix to the 96-well plate sequentially and incubate at room temperature for 60 minutes. Add ADP-Glo​^TM^ to consume the remaining ATP and incubate at room temperature for 40 minutes; then, add Kinase Detection Reagent and incubat at room temperature for 30 minutes. Finally, the multifunctional microplate reader SpectraMax i3 was used to record the luminescence.

**Isolation of the chromatin fraction**

The protocol for the cellular fractionation assay is briefly described according to existing reports [5]. Cells were collected and lysed with cytoplasmic buffer A [10 mM HEPES (pH 7.9), 0.34 M sucrose, 1.5 mM MgCl_2_, 10 mM KCl, 1 mM dithiothreitol (DTT) and 0.1% Triton X-100] containing protease inhibitor cocktail for 10 min on ice. The supernatant was removed after centrifugation at 14000 rpm for 30 min at 4°C. The cell pellet was lysed with buffer B (0.2 mM EGTA, 3 mM EDTA and 1 mM DTT) containing protease inhibitor cocktail for 30 min on ice. The chromatin pellet was obtained by centrifugation at 14000 rpm for 10 min at 4°C and boiled with SDS loading buffer for 5 min at 97°C. The samples were then subjected to Western blotting.

**Immunohistochemical analyses**

Tissue microarrays containing samples of precancerous gastric lesions (ST8017a) were purchased from Alenabio (Xian, China). Immunohistochemical analyses were performed as described [6]. The areas of positive staining were measured using Image-Pro Plus 6.0 software.

**References**

1 T Li, Ms Hung, Y Wang, Jh Mao, Jl Tan, K Jahan *et al*. Transgenic mice for cre-inducible overexpression of the Cul4A gene. *Genesis* 2011; 49: 134-141.

2 Wang Y., Zhang P., Liu Z., Wang Q., Wen M., Wang Y. *et al*. CUL4A overexpression enhances lung tumor growth and sensitizes lung cancer cells to erlotinib via transcriptional regulation of EGFR. *Molecular cancer* 2014; 13: 252.

3 Wang Y., Wen M., Kwon Y., Xu Y., Liu Y., Zhang P. *et al*. CUL4A induces epithelial-mesenchymal transition and promotes cancer metastasis by regulating ZEB1 expression. *Cancer research* 2014; 74: 520-531.

4 Olsen B. B., Issinger O. G., Guerra B. Regulation of DNA-dependent protein kinase by protein kinase CK2 in human glioblastoma cells. *Oncogene* 2010; 29: 6016-6026.

5 Hossain M. B., Shifat R., Johnson D. G., Bedford M. T. TIE2-mediated tyrosine phosphorylation of H4 regulates DNA damage response by recruiting ABL1. *Science advances* 2016; 2: e1501290.

6 Liu X., Bi L., Wang Q., Wen M., Li C., Ren Y. *et al*. miR-1204 targets VDR to promotes epithelial-mesenchymal transition and metastasis in breast cancer. *Oncogene* 2018; 37: 3426-3439.

**
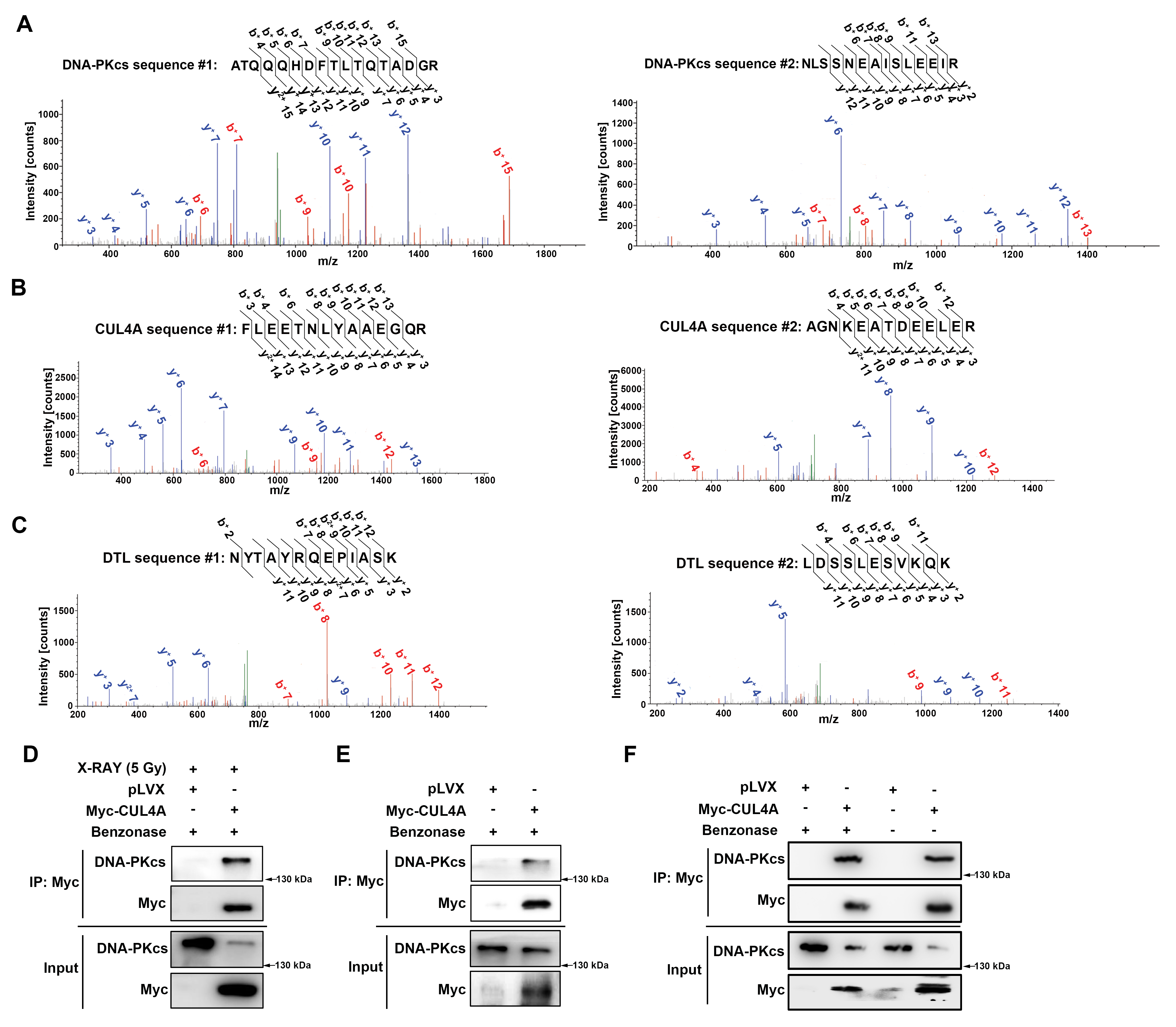
**

**Supplementary Figure 1.** Mass spectrometry identification of CUL4A interacting proteins. (A) Map of mass spectrometry peaks for DNA-PKcs interacting with HA-CUL4A; (B) Map of mass spectrometry peaks for the CUL4A sequence; (C) Map of mass spectrometry peaks for the DTL sequence; (D, E) A nuclease (Benzonase) was used to remove DNA, and coimmunoprecipitation was performed to detect the binding of CUL4A and DNA-PKcs in the presence and absence of DNA damage. (F) Coimmunoprecipitation to detect the interaction between CUL4A and DNA-PKcs in the presence or absence of Benzonase.

**
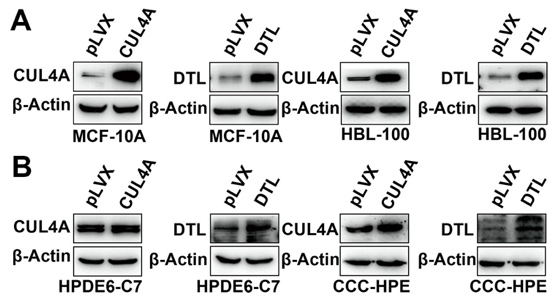
**

**Supplementary Figure 2.** Verification of cell lines with ectopic expression of CUL4A or DTL. (A) The normal breast cell lines MCF-100 and HBL-100 were used to construct cell lines with stable expression of CUL4A or DTL; (B) The normal pancreatic cell lines HPDE6-C7 and CCC-HPE were used to construct cell lines with stable expression of CUL4A or DTL. All Western blot experiments were repeated independently three times.


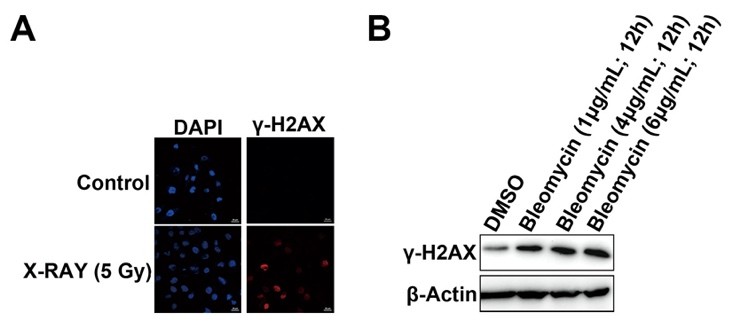


**Supplementary Figure 3.** Verification of DNA DSB induction. (A) The immunofluorescence assay showed that IR (5 Gy) induced DSBs in MCF-10A cells; (B) Western blot analysis showed that 1 μg/ml, 4 μg/ml, and 6 μg/ml bleomycin induced DSBs. All experiments were repeated independently three times. The scale bars indicate 20 μm in A.


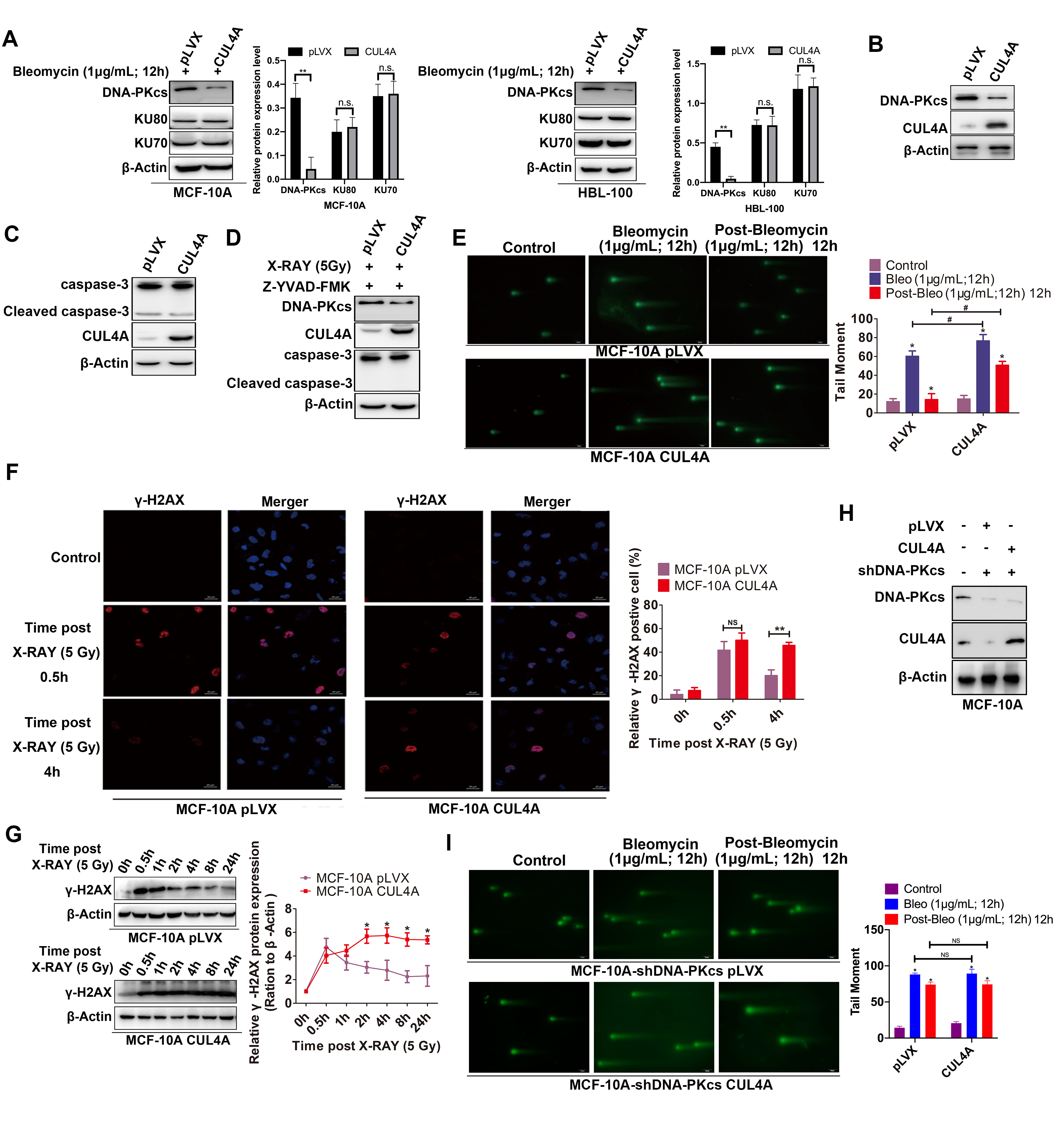


**Supplementary Figure 4.** CUL4A increased the accumulation of DSBs in normal pancreatic epithelial cells. (A) DNA-PK kinase (DNA-PKcs, KU70/80) expression in MCF-10A and HBL-100 cells with CUL4A overexpression after induction of DSBs with bleomycin; (B) DNA-PKcs expression in MCF-10A cells with or without DSBs; (C) Western blot analysis of caspase-3/cleaved caspase-3 levels in MCF-10A cells with overexpression of CUL4A; (D) MCF-10A cells overexpressing CUL4A with DNA damage were treated with a pancaspase inhibitor (Z-YVAD-FMK), and the protein expression level of DNA-PKcs was analyzed by Western blotting; (E) After treatment with bleomycin, a neutral comet assay was performed to analyze the accumulation of DSBs in MCF-10A cells overexpressing CUL4A; (F) Western blot analysis of γ-H2AX protein expression levels in MCF-10A cells overexpressing CUL4A after IR (5 Gy); (G) The percentage of γ-H2AX-positive cells among MCF-10A cells overexpressing CUL4A was analyzed by immunofluorescence after IR (5 Gy); (H) Western blot analysis of DNA-PKcs and CUL4A protein expression levels in MCF-10A cells with CUL4A overexpression and DNA-PKcs knockout; (I) A neutral comet assay was performed to analyze the accumulation of DSBs in MCF-10A cells with CUL4A overexpression and DNA-PKcs knockout. * *p*< 0.05, ** *p*< 0.01, # *p*< 0.05, NS = not significant based on Student’s *t*-test. The data are presented as the means ± SDs of three (A to F) independent experiments. The scale bars indicate 50 μm in E and I and 20 μm in F. More than 100 cells were counted in E, F and I.


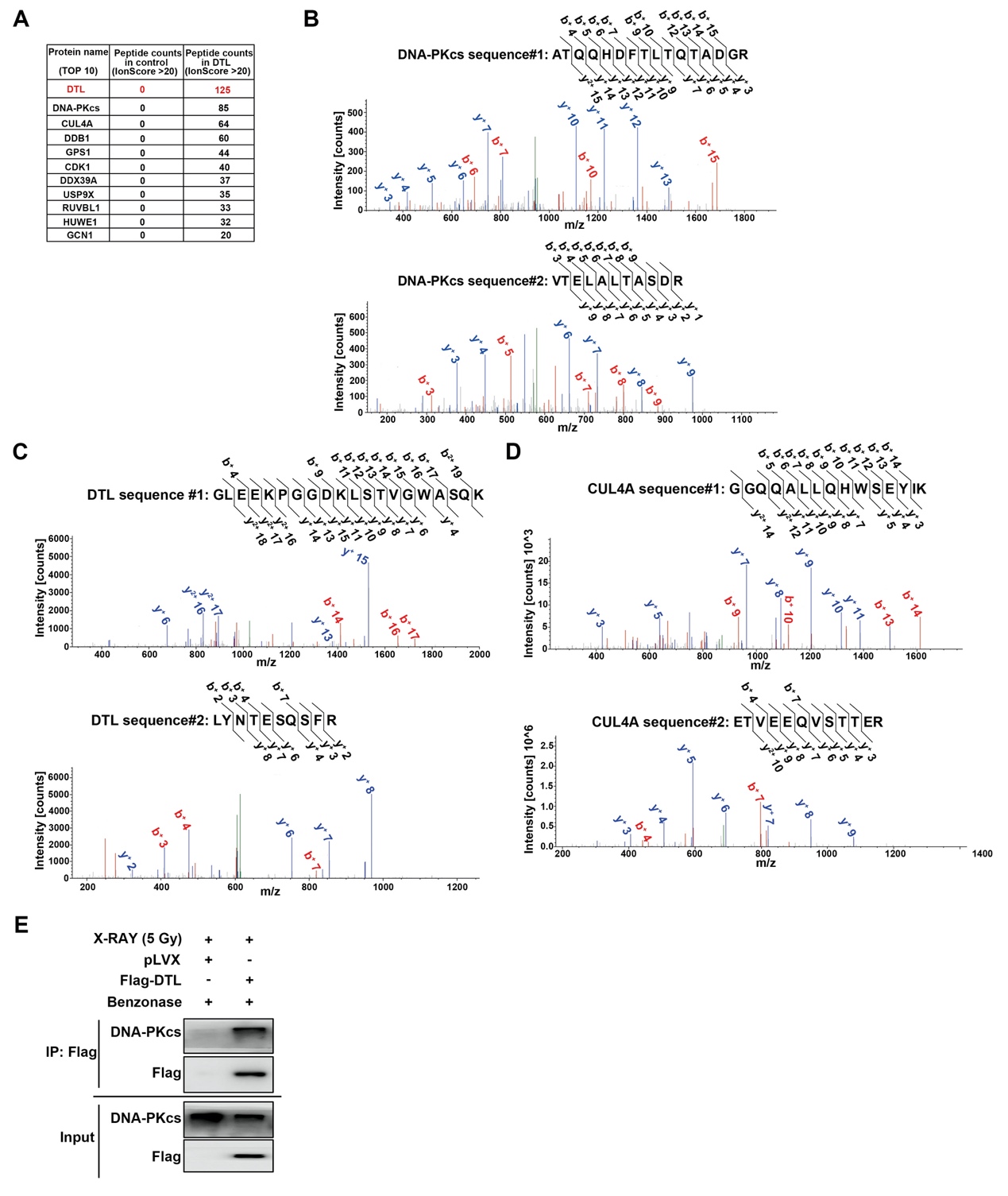


**Supplementary Figure 5.** Identification of DTL interacting proteins by mass spectrometry. (A) The top 10 abundant proteins (IonScore > 20) interacting with DTL identified by mass spectrometry; (B) Map of mass spectrometry peaks for DNA-PKcs interacting with Flag-DTL; (C) Map of mass spectrometry peaks for the DTL sequence; (D) Map of mass spectrometry peaks for the CUL4A sequence; (E) A nuclease (Benzonase) was used to remove DNA. Coimmunoprecipitation was performed to detect the binding of CUL4A and DNA-PKcs in the presence of DNA damage.


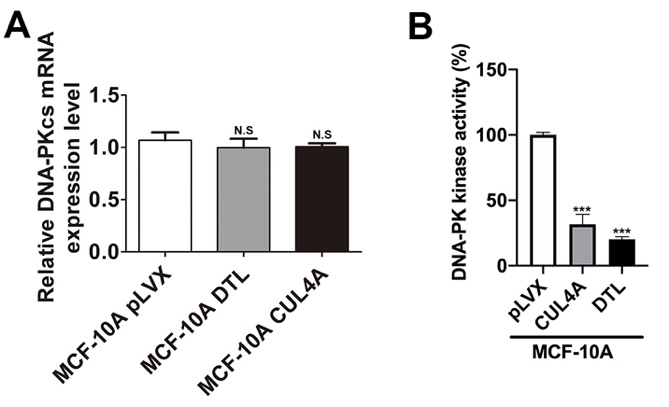


**Supplementary Figure 6.** (A) Real-time quantitative PCR results showed that neither CUL4A nor DTL affected the DNA-PKcs mRNA level; (B) DNA-PK kinase activity was detected in MCF-10A cells overexpressing CUL4A or DTL. *** *p*< 0.001, NS = not significant based on Student’s *t*-test. The data are presented as the means ± SDs of three (A and B) independent experiments.


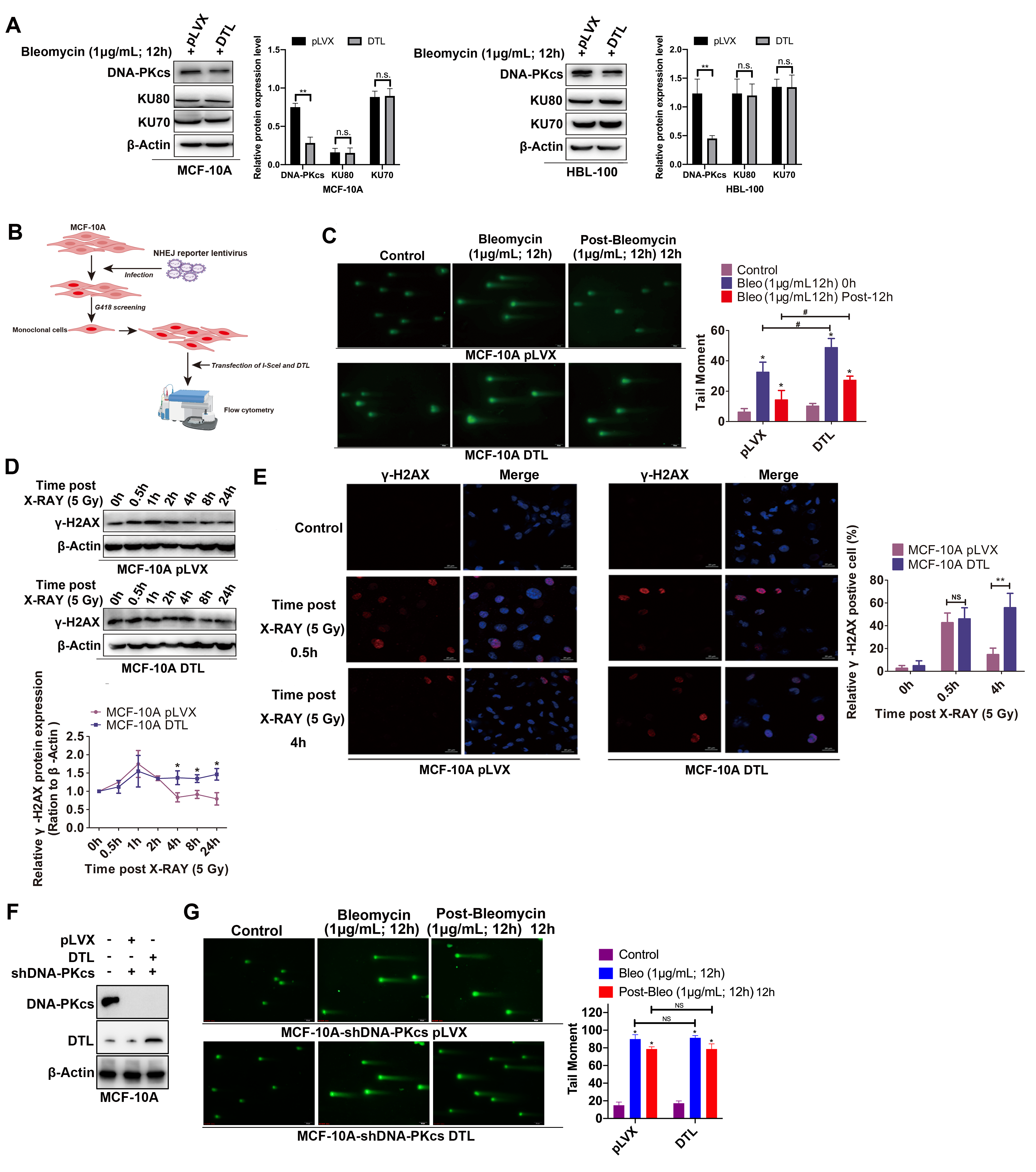


**Supplementary Figure 7.** DTL increased the accumulation of DSBs in normal pancreatic epithelial cells. (A) DNA-PK kinase (DNA-PKcs, KU70/80) expression in MCF-10A and HBL-100 cells overexpressing DTL after bleomycin treatment; (B) Schematic diagram of establishment of stable NHEJ reporter gene expression in MCF-10A cells; (C) After treatment with bleomycin, a neutral comet assay was performed to analyze the accumulation of DSBs in MCF-10A cells overexpressing DTL; (D) Western blot analysis of the γ-H2AX protein expression level in MCF-10A cells overexpressing DTL after IR; (E) The percentage of γ-H2AX-positive cells among MCF-10A cells overexpressing DTL was analyzed by immunofluorescence after IR; (F) Western blot analysis of DNA-PKcs and DTL protein expression levels in MCF-10A cells with DTL overexpression and DNA-PKcs knockout; (G) A neutral comet assay was performed to analyze the accumulation of DSBs in MCF-10A cells with DTL overexpression and DNA-PKcs knockout. * *p*< 0.05, ** *p*< 0.01, # *p*< 0.05 and NS = not significant based on Student’s *t*-test. The data are presented as the means ± SDs of three (A, C to G) independent experiments. More than 100 cells were counted in C, E and G. The scale bars indicate 50 μm in C and G and 20 μm in E.


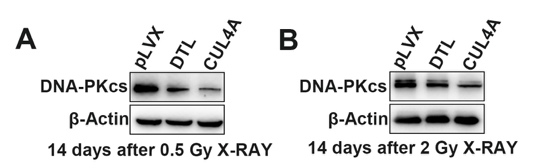


**Supplementary Figure 8.** CUL4A/DTL degraded the DNA-PKcs protein in cells cultured for 14 days after IR. (A) Western blot analysis of DNA-PK expression in cells cultured for 14 days after IR (0.5 Gy); (B) Western blot analysis of DNA-PK expression in cells cultured for 14 days after IR (2 Gy).


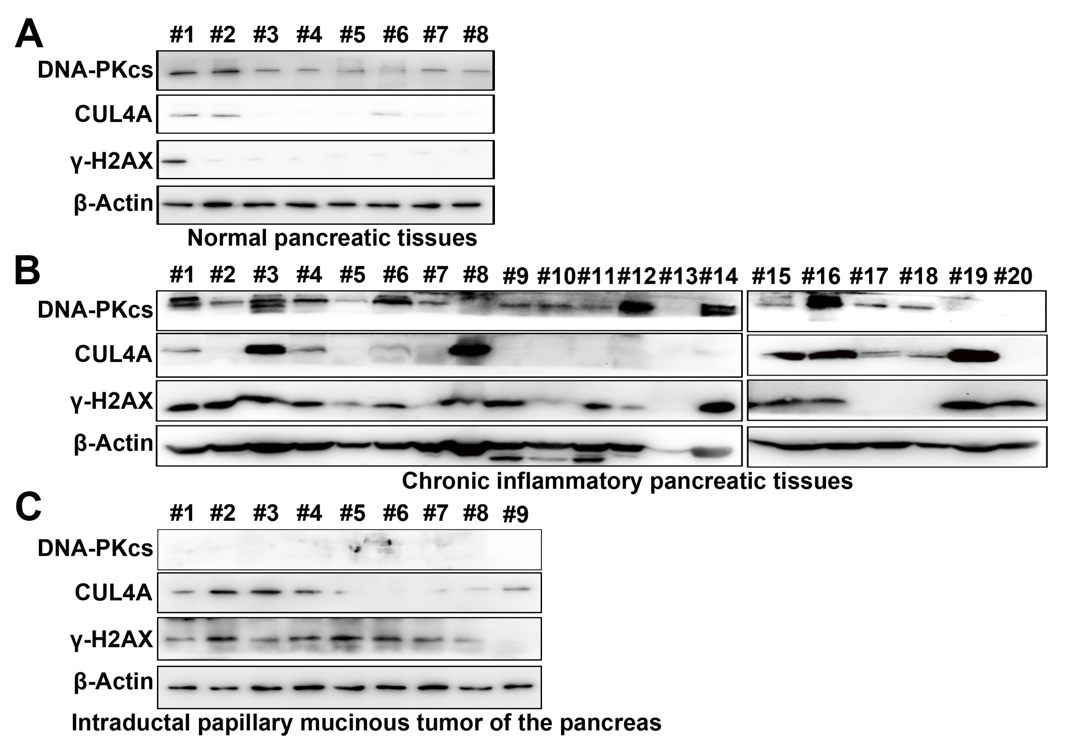


**Supplementary Figure 9.** Western blot analysis of CUL4A, DNA-PKcs and γ-H2AX protein expression levels in precancerous pancreatic tissues. The protein expression levels of CUL4A, DNA-PKcs, and γ-H2AX were detected by Western blotting in normal pancreatic tissues (A), chronically inflamed pancreatic tissues (B), and intraductal papillary mucinous neoplasm tissues (C).

**Supplementary Table 1** Mass spectrometric identification of the proteins bound to CUL4A.

| UniProtKB | GENE | UniProtKB | GENE |
| --- | --- | --- | --- |
| P55265 | ADAR | P25205 | MCM 3 |
| O95831 | AIFM 1 | P33993 | MCM7 |
| Q9C0C7 | AMBRA 1 | Q9NU22 | MDN1 |
| P48444 | ARCN1 | P43246 | MSH2 |
| Q9NVI7 | ATAD3A | O43795 | MYO1B |
| P16615 | ATP2A2 | P55786 | NPEPPS |
| P27708 | CAD | Q08J23 | NSUN2 |
| Q86VP6 | CAND1 | Q92621 | NUP205 |
| Q99459 | CDC5L | Q99623 | PHB2 |
| Q14008 | CKAP 5 | Q15149 | PLEC |
| P53618 | COPB 1 | P78527 | DNA-PKcs |
| P35606 | COPB 2 | O94906 | PRPF6 |
| Q13619 | CUL4A | Q9Y520 | PRRC2C |
| Q9Y4B6 | DCAF 1 | P49792 | RANBP2 |
| Q8TEB1 | DCAF11 | Q9Y265 | RUVBL1 |
| Q16531 | DDB1 | F1T0I1 | SEC16A |
| Q9Y2L1 | DIS3 | Q8NC51 | SERBP1 |
| P50570 | DNM 2 | Q9UJS0 | SLC25A13 |
| Q9NZJ0 | DTL | Q14683 | SMC1A |
| Q15029 | EFTUD2 | O95347 | SMC2 |
| Q96CS3 | FAF 2 | P18583 | SON |
| P22102 | GART | P19623 | SRM |
| Q92616 | GCN1 | B7Z645 | SYNCRIP |
| Q13098 | GPS1 | P0CG48 | UBC |
| Q9P035 | HACD3 | Q93008 | USP9X |
| P16403 | HIST1-HC | O14980 | XPO1 |
| Q14527 | HLTF | Q9Y5A9 | YTHDF 2 |
| Q1KMD3 | HNRNPUL2 | P31946 | YWHAB |
| P0DMV8 | HSPA1A | P56192 | MARS |
| Q7Z6Z7 | HUWE1 |  |  |
| O00425 | IGF2BP3 |  |  |
| Q12906 | ILF3 |  |  |
| O95373 | IPO7 |  |  |
| Q96P70 | IPO9 |  |  |
| O14654 | IRS4 |  |  |
| Q53G59 | KLHL12 |  |  |
| Q9P2J5 | LARS |  |  |
| P42704 | LRPPRC |  |  |

**Supplementary Table 2** Mass spectrometric identification of the proteins bound to DTL.

| UniProtKB | GENE |
| --- | --- |
| O95831 | AIFM1 |
| Q9NVI7 | ATAD3A |
| P50990 | CCT8 |
| P06493 | CDK1 |
| Q13619 | CUL4A |
| Q8NI60 | COQ8A |
| Q16531 | DDB1 |
| O00148 | DDX39A |
| Q9NZJ0 | DTL |
| Q14204 | DYNC1H1 |
| P49327 | FASN |
| P21333 | FLNA |
| Q92616 | GCN1 |
| Q13098 | GPS1 |
| Q86YZ3 | HRNR |
| P11142 | HSPA8 |
| P04792 | HSPB1 |
| Q7Z6Z7 | HUWE1 |
| P12268 | IMPDH2 |
| Q9NU22 | MDN1 |
| P78527 | DNA-PKcs |
| P62191 | PSMC1 |
| P49792 | RANBP2 |
| Q07020 | RPL18 |
| Q9Y265 | RUVBL1 |
| F1T0I1 | SEC16A |
| P53007 | SLC25A1 |
| Q02978 | SLC25A11 |
| P05141 | SLC25A5 |
| P12236 | SLC25A6 |
| O75643 | SNRNP200 |
| O95793 | STAU1 |
| O60506 | SYNCRIP |
| Q93008 | USP9X |
| P54577 | YARS1 |
| P27348 | YWHAQ |
